# The contribution of family medicine to African health systems

**DOI:** 10.4102/phcfm.v8i1.1251

**Published:** 2016-08-25

**Authors:** Robert Mash

**Affiliations:** 1 Family Medicine and Primary Care, Stellenbosch University, South Africa

Effective health care is one of the top 10 changes that people would like to see in countries such as South Africa.^1^ African health systems, however, face an enormous burden of disease that may include communicable diseases such as HIV, TB and malaria; interpersonal violence and trauma; deaths from avoidable maternal and child-related conditions; as well as a growing burden from non-communicable diseases such as hypertension and diabetes.^2^ At the same time, Africa has the fewest health care workers and often invests poorly in health systems outside the central referral hospitals.^2^

Health systems have been criticised for their reinforcement of the inverse care law – those with the greatest health needs and fewest resources have the least access to health care.^3^ Some health systems create further impoverishment through the need for out-of-pocket payments or only offer fragmented care through a selection of vertical programmes for specific priority diseases.^3^ Care may be unsafe with high risk of iatrogenic harm and may be misdirected towards treatment and curative care with little attention to health promotion and disease prevention.^3^

Countries with strong primary health care systems have better outcomes, increased patient satisfaction, less hospitalisation and lower costs.^4^ Strong primary health care systems focus on accessibility, continuity of care, co-ordination of care and a comprehensive service.^5^ Nevertheless, primary health care has also been heavily criticised in low- and middle-income countries.^3^ Primary health care often only focuses on a few priority diseases with care delivered by low level or poorly trained health workers in health posts that are marginalised and disempowered in relation to the rest of the health system.^3^ There is inequity by disease with certain diseases addressed strongly through donor-funded vertical programmes, while people’s health care needs as a whole are often unaddressed. The WHO have recommended four reforms of health systems to enable them to benefit from effective primary health care:^3^ to improve health equity by strategies to ensure universal health coverage (e.g. South Africa is pursuing national health insurance), to improve health systems by building them around the needs of people rather than diseases (being more people-centred), to improve the competency and reliability of health leadership, and to move from a focus on individual patients to policies that also promote and protect the health of communities (community-orientated primary care).

African health care systems typically rely on community health workers, clinical officers (mid-level doctors) or clinical nurse practitioners to deliver primary health care. Effective primary health care systems in countries such as Brazil or Cuba, as well as in high-income countries, include a doctor in the primary health care team – especially one with postgraduate training as a family physician.^6^ A family physician typically has 4-years of postgraduate training to become an expert generalist. Health care systems in high-income countries have shown a correlation between the supply of family physicians and reduced morbidity, mortality, increased life expectancy, lower costs and improved outcomes.^4^ Countries such as South Africa are beginning to realise the importance of doctors in the primary care team and now have a goal that every clinic should have access to a doctor.^7^ The World Health Assembly has recommended that family physicians are an essential part of the multidisciplinary primary health care team.^8^

Africa is the one continent in the world that has not yet embraced the training of family physicians for their health care systems.^9^ In the African context, the family physician is trained to work clinically in the community, in primary care and in the generalist hospital within the district health services.^10^ This generalist hospital may have different names (district hospital, primary hospital and sub-district hospital), but is characterised by the fact that services are not offered through specialist departments but by generalists across wards that are often just labelled as male, female, child and maternity. The African family physician, especially in rural areas, will therefore need procedural skills appropriate to this type of hospital – obstetric, anaesthetic and surgical skills.^10^ Unfortunately, the hospital-centred orientation of African health systems and medical schools often leads to family physicians being inappropriately placed at central or tertiary hospitals, where they struggle to make a difference in the shadow of all the other specialist departments.

Family physicians also bring a range of skills to support the primary care team.^10^ They will be a consultant to the primary care team who sees more complicated patients, who is a role model of patient-centred care and who can mentor and build capacity in the other team members. They bring skills in improving the quality of care (clinical governance) and a level of critical thinking that will enable the team to make sense of the health needs of the community and to respond appropriately (community-orientated primary care). The family physician may move between the community health teams, the primary care facility and the hospital. In Africa, it will, of necessity, be a multidisciplinary team approach to primary health care.

In countries that are contemplating the placement of family physicians, there are several myths that need to be addressed:

‘A family physician is constructed from the parts of other specialists’. In other words a family physician is really just a combination of a mini-paediatrician, mini-obstetrician, mini-surgeon and mini-physician. One specialist in Rwanda stated it would take 20 years to train such a person if they were to be competent in all these areas. It should be clear from the description above that the role of the family physician is tailored to the context of the district health services, and while they may share some skills with other specialists, they have a different role to play in the health system to the referral hospital-based specialist.

‘A family physician is useful to fill gaps in the health system’. One of the risks of training a competent generalist is that they can be deployed to fill gaps in the referral hospital when the specialist is absent. Family physicians end up working as hospital specialists and upskilling themselves to perform this role. Unfortunately, this means that they are no longer fulfilling their role as a generalist in the district health services.

‘Family physicians are specialists and not generalists’. Family physicians are registered as specialists in their field because they have completed the same amount of post-graduate training as the hospital specialists. If we describe ourselves as specialists, however, we confuse the health system and the managers who are used to the work of specialists in referral hospitals. For example, in South Africa family medicine was initially considered as a sub-speciality of medicine by human resources, who worked out that we needed more ophthalmologists than family physicians. District health services were also forbidden to appoint specialists at one point which led to family physicians being appointed inappropriately at referral hospitals. Although we are trained in the same model as specialists, we are trained as expert generalists.

‘Family physicians are trained to be family physicians at medical school’. A widely held view is that newly qualified doctors are competent generalists. In most African countries, no further training is required. It should be clear, however, from the description above that medical school produces a competent basic doctor who is ready for internship and further development – but not a competent family physician.

‘Family physicians are managers and not clinicians’. In the district health services, there is often a deficiency of managerial capacity. It is tempting for the family physician to be drawn into this vacuum and to take responsibility for corporate governance (supply chain, human resources, finances and infrastructure). All too quickly you can end up attending management meetings and being divorced from the clinical role for which you trained. While family physicians may take responsibility for clinical governance (the quality of clinical services), there is a need for effective clinical managers to handle corporate governance.

Family physicians therefore have an essential role to play in strengthening primary health care and district health services in the African context. Their role in the African context may differ from general practitioners and family physicians in more highly resourced settings. Countries in Africa that are committed to primary health care should invest in the training of family physicians as one of the key members of the primary health care team and district health services.
